# Recurrent C3 Glomerulonephritis along with BK-Virus-Associated Nephropathy after Kidney Transplantation: A Case Report

**DOI:** 10.3390/medicina59071308

**Published:** 2023-07-14

**Authors:** Jeong-Hoon Lim, Seong-Won Shin, Mee-Seon Kim, Man-Hoon Han, Yong-Jin Kim, Hee-Yeon Jung, Ji-Young Choi, Jang-Hee Cho, Sun-Hee Park, Yong-Lim Kim, Deokbi Hwang, Woo-Sung Yun, Hyung-Kee Kim, Seung Huh, Eun Sang Yoo, Dong Il Won, Chan-Duck Kim

**Affiliations:** 1 Department of Internal Medicine, School of Medicine, Kyungpook National University Hospital, Kyungpook National University, Daegu 41944, Republic of Korea; jh-lim@knu.ac.kr (J.-H.L.); kei010501@naver.com (S.-W.S.); hy-jung@knu.ac.kr (H.-Y.J.); jyss1002@hanmail.net (J.-Y.C.); jh-cho@knu.ac.kr (J.-H.C.); sh-park@knu.ac.kr (S.-H.P.); ylkim@knu.ac.kr (Y.-L.K.); 2 Department of Pathology, School of Medicine, Kyungpook National University Hospital, Kyungpook National University, Daegu 41944, Republic of Korea; kimm23@naver.com (M.-S.K.); one-many@hanmail.net (M.-H.H.); yyjjkim@knu.ac.kr (Y.-J.K.); 3 Department of Surgery, School of Medicine, Kyungpook National University Hospital, Kyungpook National University, Daegu 41944, Republic of Korea; db.surlife@gmail.com (D.H.); wsyun@me.com (W.-S.Y.); hkkim6260@knu.ac.kr (H.-K.K.); shuh@knu.ac.kr (S.H.); 4 Department of Urology, School of Medicine, Kyungpook National University Hospital, Kyungpook National University, Daegu 41944, Republic of Korea; uroyoo@knu.ac.kr; 5 Department of Clinical Pathology, School of Medicine, Kyungpook National University Hospital, Kyungpook National University, Daegu 41944, Republic of Korea; wondi@knu.ac.kr

**Keywords:** C3 glomerulopathy, C3 glomerulonephritis, kidney transplantation, graft biopsy, BK virus

## Abstract

C3 glomerulonephritis (C3GN) is a rare cause of end-stage kidney disease and frequently recurrent in allografts following kidney transplantation (KT). Herein, we describe the case of a kidney transplant recipient who developed recurrent C3GN along with BK-virus-associated nephropathy (BKVAN) following KT. A 33-year-old man diagnosed with membranoproliferative glomerulonephritis 17 years ago underwent preemptive KT with a donor kidney from his aunt. Proteinuria gradually increased after 3 months following KT, and graft biopsy was performed 30 months after KT. Histopathological examination revealed recurrent C3GN. The dosages of triple immunosuppressive maintenance therapy agents were increased. Subsequently, serum C3 levels recovered to normal levels. However, at 33 months following KT, the BK viral load increased and graft function gradually deteriorated; a second graft biopsy was performed at 46 months following KT, which revealed BKVAN and decreased C3GN activity. The dosages of immunosuppressive agents were decreased; subsequently, BKVAN improved and graft function was maintained with normal serum C3 levels at 49 months following KT. This case indicates that C3GN is highly prone to recurrence following KT and that immunosuppressive therapy for C3GN increases the risk of BKVAN.

## 1. Introduction

C3 glomerulopathy (C3G) is a clinicopathologic disease entity caused by dysregulation of the alternative complement pathway in plasma and the glomerular microenvironment [1]. In 2012, experts in renal pathology, nephrology, and complementology held a consensus conference on C3G [2]. C3G is characterized by histopathological findings which show accumulation of the C3 component of complement in kidney tissue [3]. C3G is a disease process secondary to abnormal control of complement activation, deposition, or degradation characterized by predominant glomerular C3 fragment deposition with electron-dense deposits on electron microscopy [3]. The incidence of C3G is very low, with estimates ranging from 1 case per 1,000,000 to 3 cases per 1,000,000, and the prevalence rates may be as low as 5 cases per 1,000,000 [4]. Because of this rarity, clinical information about C3G is sparse.

C3G is divided into C3 glomerulonephritis (C3GN) and dense-deposit disease (DDD) based on the location of glomerular deposits on electron microscopy images. DDD frequently reveals electron-dense, osmiophilic deposits with a sausage-shaped appearance that thicken and transform the lamina densa of the glomerular basement membrane (GBM) [3]. C3GN is a subtype of C3G without the characteristic appearances of DDD. C3G has a high recurrence rate in kidney transplant recipients (KTRs); however, owing to its rarity, clinical information on this topic is limited. Additionally, there are no reports of BK virus-associated nephropathy (BKVAN) during the treatment of recurrent C3GN. In this report, we present a case of a KTR with recurrent C3GN and BKVAN.

## 2. Case Presentation

**Figure 1:**
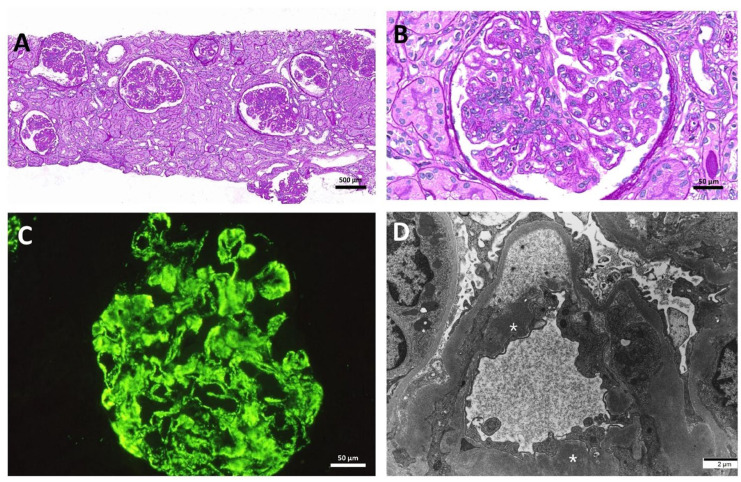
Native kidney histopathological findings. (**A**) Glomeruli showing proliferative changes along with a non-inflamed interstitium (PAS; original magnification ×200). (**B**) A representative glomerulus showing lobular accentuation with mesangial cell proliferation (PAS; original magnification ×400). (**C**) Positive staining for C3 antibody along the capillary wall and the mesangial area with a granular pattern (immunofluorescence microscopy; original magnification ×400). (**D**) Electron-dense deposits are located in the subendothelial area and mesangium (original magnification ×5000). Asterisks indicate electron-dense deposits. Abbreviation: PAS, periodic acid–Schiff.

A 33-year-old Korean male with end-stage kidney disease (ESKD) underwent preemptive living-donor kidney transplantation (KT) from his aunt. At the age of 16, he was diagnosed with type I membranoproliferative glomerulonephritis following kidney biopsy, and C3 deposition was detected by immunofluorescence microscopy (Figure 1).

At that time, the serum C3 level was low (12.0 mg/dL; reference: 90–180 mg/dL), which meets the diagnostic criterion for C3GN; however, the concept of C3G had not been established. He received immunosuppressive therapies, but his kidney function gradually deteriorated and progressed to ESKD. On pretransplant evaluation, panel-reactive antibodies were not detected, and cross-matches were negative. The recipient’s blood type was O and that of the donor was B; therefore, he received pretransplant desensitization for ABO incompatibility. The initial anti-ABO IgG titer was 1:64; four sessions of therapeutic plasma exchange (TPE) and 200 mg of rituximab were administered [4]. Following desensitization, the anti-ABO IgG titer decreased to 1:4 at the time of transplantation. The patient initially received standard triple immunosuppressive therapy with tacrolimus 6 mg/day, mycophenolate sodium 1080 mg/day, and methylprednisolone 500 mg/day. No acute complications were noted following KT, and the patient was discharged 14 days later.

**Figure 2:**
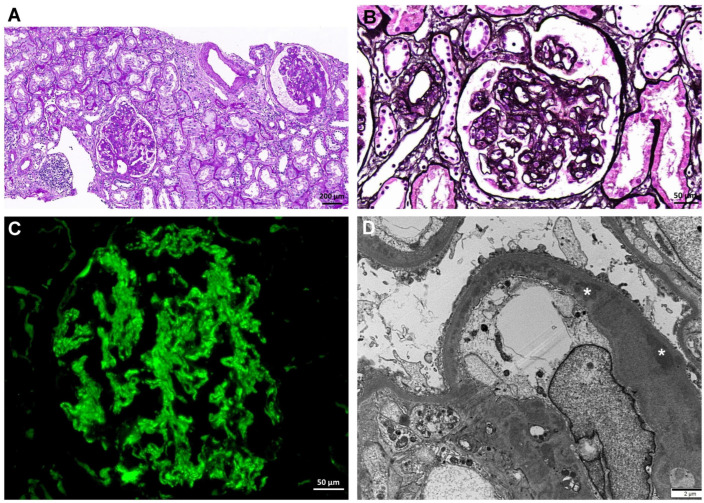
Graft histopathological findings at 30 months following KT. (**A**) Glomeruli showing proliferative changes. The interstitium has less inflammatory cell infiltration (PAS; original magnification ×200). (**B**) A representative glomerulus showing the lobular accentuation and focal doubling of the capillary wall (PAM; original magnification ×400). (**C**) C3 antibody is observed along the capillary wall and the mesangial area with a granular pattern (immunofluorescence microscopy; original magnification ×400). (**D**) Electron-dense deposits are located in the capillary basement membrane and mesangium (original magnification ×5000). Asterisks indicate electron-dense deposits. Abbreviations: KT, kidney transplantation; PAS, periodic acid–Schiff; PAM, periodic acid methenamine silver.

Three months later, increased proteinuria was noted; at 12 months following KT, the spot urine protein-to-creatinine ratio was 2.3 g/g. Proteinuria was treated with angiotensin receptor blockers and the level was decreased less than 1.0 g/g. Thirty months following KT, spot urine protein-to-creatinine ratio increased to 1.4 g/g, so graft biopsy was performed (Figure 2). The graft biopsy revealed glomerular lobular accentuation and focal doubling of the capillary wall. Immunofluorescence microscopy revealed C3 antibodies; C3 staining was at least two grades stronger than any combination of C1q, C4, IgG, IgM, and IgA.

**Figure 3:**
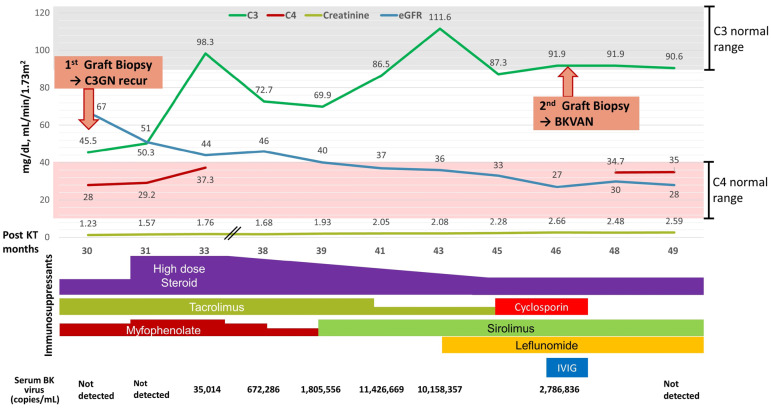
Clinical course and treatment. Abbreviations: C3GN, C3 glomerulonephritis; eGFR, estimated glomerular filtration rate; KT, kidney transplantation; BKVAN, BK-virus-associated nephropathy; IVIG, intravenous immunoglobulin.

Electron-dense deposits were noted in the capillary basement membrane and mesangium. The serum C3 level was 45.5 mg/dL. In the genetic tests, there were no variants in the *C3*, *CFB*, *CFH*, *CFI*, and *CFHR1-5*. Based on these findings and previous history, the patient was diagnosed with C3GN recurrence, and the dosages of immunosuppressive agents were increased. The methylprednisolone dose was increased from 4 mg/day to 40 mg/day and that of mycophenolate sodium was increased from 720 mg/day to 1080 mg/day. After 3 months of treatment, the serum C3 level recovered to 98.3 mg/dL, and the BK viral load increased to 3.5 × 10^4^ copies/mL. Consequently, tacrolimus was replaced by cyclosporine, mycophenolate sodium was changed to sirolimus, and leflunomide was added. Cyclosporine trough level was maintained between 80 and 100 ng/mL. At 46 months following KT, the plasma BK viral load decreased from 1.0 × 10^7^ copies/mL to 2.8 × 10^6^ copies/mL, and the serum creatinine level increased to 2.7 mg/dL; serum C3 levels remained within the normal range (Figure 3).

**Figure 4:**
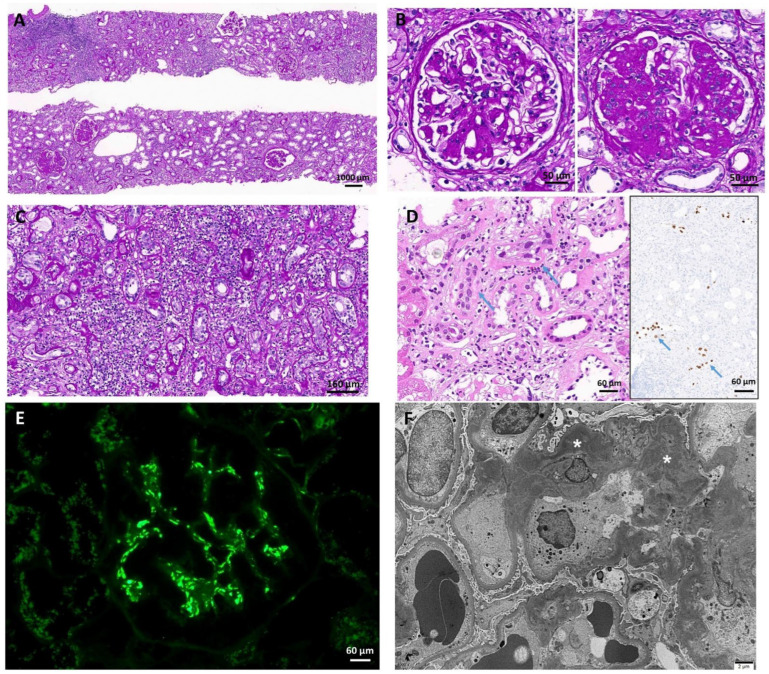
Graft histopathological findings at 46 months following KT. (**A**) The upper core showing massive interstitial inflammation in the deep cortex. The lower core of the upper cortex showing proliferative glomeruli and mild interstitial inflammation (PAS; original magnification ×40). (**B**) The left glomerulus showing mesangial expansion with mild cellular proliferation. The right glomerulus has sclerotic changes. Both patterns are mixed with variable degrees in other glomeruli (PAS; original magnification ×400). (**C**) High-power view of the most severe interstitial inflammatory area. Some tubules are infiltrated by lymphocytes and have viral inclusion-like nuclei (PAS; original magnification ×200). (**D**) Left: BK-virus-infected tubular nuclei of tubular epithelium (blue arrows; PAS; original magnification ×400). Right: SV40 antibodies are present in tubular epithelial nuclei (blue arrows; original magnification ×200). (**E**) C3 antibodies are noted in the mesangial area with a granular pattern. C3 antibody positivity in the capillary wall is minimal (immunofluorescence microscopy; original magnification ×200). (**F**) Electron-dense deposits are absent in the membrane and present in the mesangium (original magnification ×5000). Asterisks indicate electron-dense deposits. Abbreviation: KT, kidney transplantation; PAS, periodic acid–Schiff.

A second graft biopsy was performed to differentiate among graft rejection, C3GN reactivation, and BKVAN (Figure 4).

Glomeruli showed mesangial expansion with mild cellular proliferation and some glomeruli revealed sclerotic changes. Massive interstitial inflammation was noted in the deep cortex, and immunohistochemistry revealed SV40 positivity in the tubular epithelial nuclei. The levels of C3 antibodies in the capillary wall decreased, and electron-dense deposits disappeared from the membrane and were located only in the mesangium. Based on the histopathological findings, new-onset BKVAN was diagnosed with decreased C3GN activity. For BKVAN treatment, cyclosporine was discontinued, and intravenous immunoglobulin (0.3 g/kg) was initiated. At 49 months following KT, the plasma BK virus load was undetectable, and the serum C3 level was normal at 90.6 mg/dL. Moreover, graft function was maintained with a serum creatinine level of 2.5 mg/dL without further deterioration.

## 3. Discussion

We presented a case of recurrent C3GN following KT with new-onset BKVAN during C3GN treatment. Despite pretransplant desensitization with TPE and rituximab administration for pretransplant ABO incompatibility, the patient developed early C3GN recurrence. After confirming C3GN recurrence, the dosages of immunosuppressive drugs were increased, which helped control the C3GN activity by increasing serum C3 levels and decreasing C3 deposition. However, to avoid complications of robust immunosuppression, including BKVAN, tailored immunosuppression and careful surveillance are required.

C3G is caused by alternative complement pathway dysregulation [1,2,5,6,7]. Autoantibodies, such as C3 convertase of the alternative pathway, and autoantibodies against factors H and B, reduce C3 degradation and increase C3 fragment deposition in the GBM, resulting in C3G [3,8,9,10]. C3G is divided into the following two categories: C3GN, characterized by electron-dense deposits in the glomerular matrix (subendothelial and a few intramembranous deposits); and DDD, characterized by highly electron-dense, osmiophilic deposits in the lamina densa of the GBM [11,12,13,14,15].

When diagnosing C3G, ruling out infection-related glomerulonephritis and post-infectious glomerulonephritis is important prior to diagnosis of C3G. Glomerulonephritis with C3-dominant deposition caused by infections can be differentiated with C3G by the history of infection and normalization of C3 level within 12 weeks. In addition, they are presumed to be non-recurrent and treatment is to resolve the infection during supporting kidney function [16]. In this case, C3 decreased even after 12 weeks, there was no history of infection, and C3 decreased with C3 deposition in the native kidney, suggesting recurrence of C3GN following KT.

Few researchers have reported on recurrent C3GN following KT (Table 1).

C3GN is a highly recurrent type of glomerulonephritis following KT, with a recurrence rate of 67–83% [5,17,18,19]. The time to recurrence was median 28 months (range, 9 days to 11 years) in C3GN [5] and 164 months (range, 9 to 207 months) in C3GN with CFHR5 nephropathy [19]. However, there is no evidence to support serum complement monitoring for predicting the risk of recurrent C3GN following KT, and there are no known strategies for reducing the risk of C3GN recurrence [20,21]. According to a recent expert conference, during the acute period of kidney loss and acute inflammation, KT should be avoided because the rapid deterioration of kidney function in native kidneys is related to an increased risk of recurrence [21].

The risk of graft failure is high in KTRs with recurrent C3GN, accounting for 30–50% of graft failure cases [5,17,22]. Although effective treatment for C3GN is unclear, various immunosuppressive therapies are used based on the pathophysiological mechanisms underlying C3GN. In addition to modulating the standard triple immunosuppressive agents, including calcineurin inhibitors, mycophenolic acids, and corticosteroids, other immunosuppressive therapies, including rituximab and eculizumab administration and TPE, have been evaluated in limited studies. A recently published systematic review analyzed the effects of C3G treatment in KTRs [22]. In 122 KTRs with C3G across 12 studies, 66 patients (38 and 28 with C3GN and DDD, respectively) did not receive C3G treatment owing to stable graft function and at the clinical discretion of the physicians. The pooled estimated rate of graft failure was 32% (95% confidence interval [CI], 7–64%) for C3GN and 53% (95% CI, 28–77%) for DDD. Of the 56 patients who received additional treatment, the pooled estimated rates of graft failure were 33% (95% CI, 12–57%) with eculizumab, 42% (95% CI, 2–89%) with TPE, and 81% (95% CI, 50–100%) with rituximab. In the subgroup analysis of C3GN (n = 35), the pooled estimated rates of graft failure were 22% (95% CI, 5–46%) with eculizumab, 56% (95% CI, 6–100%) with TPE, and 70% (95% CI, 24–100%) with rituximab. We did not evaluate the detailed information of recurrent C3GN using genetics or functional complement studies, because the increase in the dosages of immunosuppressive agents without using anticomplement therapy was effective in our patient. If severe C3GN is irresponsive to immunosuppressive agents, genetic and functional complement studies, and anticomplement therapy, including the use of eculizumab, should be considered.

Owing to the risk of infection, high-intensity immunosuppression has both positive and negative effects. BKVAN is one of the leading causes of graft failure in KTRs, and timely diagnosis and reduction of immunosuppression are crucial [23,24]. In this case, BKVAN occurred following an increase in the dosages of maintenance immunosuppressive agents without additional anticomplement therapy. Therefore, if the intensity of immunosuppression is increased for C3GN treatment, careful surveillance for BK virus replication and timely reduction of the dosages of immunosuppressive agents are required. Furthermore, if the graft function deteriorates, BKVAN should be considered.

C3GN is highly prone to recurrence following KT; therefore, careful observation is required during the follow-up. Increasing the dosages of maintenance immunosuppressive agents reduces C3GN disease activity but increases the risk of BKVAN. Therefore, appropriate immunosuppression modulation considering the patient’s condition is required.

## Figures and Tables

**Table 1:** Summary of studies on C3 glomerulonephritis in kidney transplant recipients.

Study	Patient Number	Country	Age at the Time of Diagnosis (Years)	Time from Diagnosis to ESKD (Months)	Time from KT to Recurrence (Months)	Median Follow-Up Duration (Months)	Treatment of Recurrent C3GN (n)	Graft Failure
Zand et al. [5] 2014	21 (recurred in 14 [66.7%])	USA	21 (range, 7–67)	42 (range, 0–400)	28 (range, 9 days to 11 years)	75.5 (range, 0–162.7)	RTX: 3 TPE: 1 No therapy: 10	7 of 14 (50.0%)
Regunathan-Shenk et al. [17] 2019	12 (recurred in 10 [83.3%])	USA	22 (range, 12–60)	48 (range, 0–190)	NA	76 (range, 9–203)	Eculizumab: 3 TPE: 1 No therapy: 7	3 of 10 (30.0%)
Bomback et al. [18] 2012	3 (recurred in 2 [66.7%])	USA	22 (range, 20–25)	123 (range, 113–134)	2.5 (range, 1–4)	13	Eculizumab: 2 TPE: 1	0
Frangou et al. [19] 2019	17 (recurred in 12 [70.6%])	Cyprus	48 (range, 32–64)	NA	164 (range, 9–207)	157 (range, 9–282)	TPE: 2 No therapy: 10	5 of 12 (41.7%)

Abbreviations: ESKD, end-stage kidney disease; KT, kidney transplantation; C3GN, C3 glomerulonephritis; RTX, rituximab; TPE, therapeutic plasma exchange; NA, not available.

## Data Availability

The original contributions presented in this study are included in the article, and further inquiries can be directed to the corresponding author.
